# Sleep apnea severity in patients undergoing atrial fibrillation ablation: Home sleep apnea‐test and polysomnography comparison

**DOI:** 10.1002/joa3.12869

**Published:** 2023-06-01

**Authors:** Nobuaki Tanaka, Masato Okada, Koji Tanaka, Toshinari Onishi, Yuko Hirao, Shinichi Harada, Masatsugu Kawahira, Yasushi Koyama, Kenshi Fujii, Heitaro Watanabe, Atsunori Okamura, Yasushi Sakata, Katsuomi Iwakura

**Affiliations:** ^1^ Cardiovascular Center Sakurabashi‐Watanabe Hospital Osaka Japan; ^2^ Department of Cardiovascular Medicine Osaka University Graduate School of Medicine Suita Japan

**Keywords:** atrial fibrillation, catheter ablation, home sleep apnea test, polysomnography, sleep apnea

## Abstract

**Background:**

Sleep apnea (SA) is highly prevalent and should be treated in patients referred for catheter ablation (CA) of atrial fibrillation (AF). Watch‐type peripheral arterial tonometry (WP) for home SA testing has demonstrated a high correlation of the apnea‐hypopnea index (AHI) with Polysomnography (PSG), but the evidence of its accuracy in AF patients is not adequate.

**Methods:**

This study was conducted under a retrospective, single‐center, observational design. We included 464 consecutive AF patients (age 65 ± 11 years, 76.5% male, 45.0% paroxysmal‐AF) who received both WP and PSG during the periprocedural period of the CA. We compared the AHI using the WP (WP‐AHI) to that using PSG (PSG‐AHI).

**Results:**

The WP‐AHI was 25.9 ± 12.7 and PSG‐AHI 31.4 ± 18.9 (*r* = .48). Among 325 patients with a WP‐AHI < 30, 116 (35.7%) exhibited a PSG‐AHI ≥ 30. Only 12.5% of the patients were indicated for continuous positive airway pressure (CPAP) treatment only by the WP‐AHI, while 70.9% were indicated for CPAP by the PSG‐AHI according to the Japanese health insurance system. The best cut‐off value of the WP‐AHI was 18.1 to predict a PSG‐AHI ≥ 20 with an area under the curve of 0.72 (95% confidence interval, 0.67–0.76).

**Conclusions:**

The WP‐AHI and PSG‐AHI were weakly correlated in AF patients receiving CA. About one‐third of the patients with moderate SA using the WP was diagnosed with severe SA evaluated by PSG. The majority required PSG for the CPAP indication.

## Graphical abstract

Only 12.5% had a CPAP indication determined by the Watch PAT‐AHI, while 70.9% did so by the PSG‐AHI according to the Japanese health insurance system.

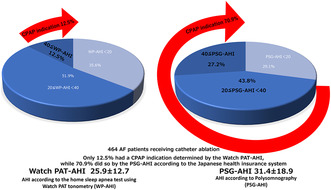

## 1 INTRODUCTION

Atrial fibrillation (AF) and sleep apnea (SA) are common, and both have a risk of cardiovascular morbidity and mortality.^1^, ^2^ SA is highly prevalent in AF patients because of the linking mechanisms between SA and AF including activation of the sympathetic nervous system during intermittent hypoxia and structural remodeling of the left atrium through the negative intrathoracic pressure during an obstructive apnea episode.^3^, ^4^

Experimental, epidemiological, and clinical studies have consistently shown that SA is a potential risk factor for the incidence and recurrence of AF.^5^ A meta‐analysis reported that obstructive SA (OSA) is associated with a 31% greater risk of AF recurrence after catheter ablation (CA) of AF.^6^ Treating OSA with continuous positive airway pressure (CPAP) reduces the AF recurrence after pulmonary vein isolation.^7^, ^8^ Nevertheless, OSA is frequently undiagnosed and consequently undertreated in AF patients receiving CA.

We previously reported that patients undergoing CA of AF had a high prevalence of SA using a watch‐type peripheral arterial tonometry (WP) device for the home sleep apnea test (HSAT) even if they did not have sleepiness or risk factors such as obesity and hypertension.^9^ Although polysomnography (PSG) is currently considered the gold standard diagnostic test for SA, carrying out PSG in all AF patients is difficult in clinical practice due to the limited access and cost issues.^10^ HSAT using WP is a simplified alternative screening tool, which can be performed in all AF patients.^11^ Although a high degree of correlation between the apnea‐hypopnea index (AHI) derived from WP (WP‐AHI) and PSG (PSG‐AHI) has been demonstrated in patients suspected of SA, the evidence of the accuracy in AF patients is not adequate.

## 2 METHODS

### 2.1 Patient population and study design

This was a retrospective, single‐center, and observational designed study to compare an automatically scored WP‐AHI with the PSG‐AHI in patients that underwent CA of AF. Out of 1435 patients who underwent CA of AF from March 2018 to December 2020 in our institute, we enrolled a total of 464 patients who received sleep studies both at home and during hospitalization including 2 patients that received alpha blockers. The HSAT was conducted using a Watch‐PAT200U (WP) (Watch‐PAT; Itamar Medical Ltd.) before the CA of AF. An in‐hospital examination was conducted using PSG after the CA of AF. We sent the WP to the patients' homes after we decided in the outpatient clinic to perform a CA of AF. The patients self‐administered the WP in their homes and returned both the WP and screening questionnaire to us before their admission for CA. We performed the PSG in a post‐operative stable condition at least 1 day after the CA of AF. During the CA procedures, most patients underwent conscious sedation with a combination of a bolus of thiamylal sodium, pentazocine, and continuous dexmedetomidine hydrochloride, except for 10 patients who underwent general anesthesia.^12^ Almost all patients received the PSG within 1 week after the CA. The mean duration between the CA and PSG was 9.3 days (median 1 day). All patients gave their informed consent for both the ablation procedures and the use of their clinical data in a retrospective study. This study complied with the Declaration of Helsinki and was performed according to the institutional ethics committee's approval.

### 2.2 Watch PAT system

The apnea‐hypopnea index measurements using the WP (WP‐AHI) consisted of the peripheral arterial tonometry (PAT) signal, oxygen saturation, heart rate, wrist activity (actigraphy), snoring, and body position. The WP indirectly detected any apnea‐hypopnea events by selectively measuring the peripheral arterial volume changes using a finger‐mounted plethysmograph. This information was collated with the pulse oximetry in conjunction with the heart rate and was further analyzed using a predeveloped automated computer program. The WP provided an algorithm able to differentiate between sleep and awake states every 30 s and to calculate both the total sleep time and total recording time. PAT‐based device determinations of the AHI (i.e., WP‐AHI) were computed automatically.

### 2.3 Polysomnography

All patients underwent overnight PSG during hospitalization using a digital polygraph system (Alice; Philips Respironics). The apnea and hypopnea events were quantified, and the SDB severity was assessed using the frequency of the apnea and hypopnea events per hour of sleep (i.e., PSG‐AHI).

### 2.4 Definitions and endpoints

The diagnosis of sleep apnea was determined according to the Adult Obstructive Sleep Apnea Task Force of the American Academy of Sleep Medicine. A normal sleep study was defined as an AHI < 5, mild sleep apnea as an AHI range ≥ 5 and <15, moderate sleep apnea as an AHI range ≥ 15 and <30, and severe sleep apnea as an AHI ≥ 30. The CPAP indications were defined as an AHI ≥ 20 assessed by PSG or an AHI ≥ 40 assessed by HSAT according to the current Japanese health insurance system. The Epworth Sleepiness Scale (ESS) is a commonly used 8‐item questionnaire to assess subjective daytime sleepiness.^13^ The ESS is unidimensional and is closely related to the frequency of apnea events during sleep apnea. The ESS scores were collected by sending a paper questionnaire to the patients' homes together with the WP before the CA. Paroxysmal AF was defined as an AF episode that terminated spontaneously or following the administration of antiarrhythmic drugs within 7 days of the onset. Persistent AF was defined as an AF episode lasting for more than 7 days and up to 1 year. Long‐lasting AF was defined as that persisting for more than 1 year.

The aim of this study was to compare the automatically scored WP‐AHI with the PSG‐AHI and to elucidate the risk of a CPAP indication assessed using PSG. Consequently, the primary endpoints of the study were (1) the distribution of the WP‐AHI and PSG‐AHI among the patients and (2) the predictors of a CPAP indication (PSG‐AHI ≥ 20) among them.

### 2.5 Statistical analysis

The descriptive statistics are reported as the mean ± SD for continuous variables and as absolute frequencies and percentages for categorical variables. The parametric data were compared using a Student's *t*‐test or paired Student's *t*‐test, and the non‐parametric data were compared using the Mann–Whitney *U*‐test, Wilcoxon signed rank test, chi‐squared test, or Fisher's exact test, as appropriate. A receiver operating characteristic (ROC) curve analysis was used to determine the optimal cut‐off value of the WP‐AHI to predict a CPAP indication assessed using PSG (AHI ≥ 20). Those cut‐off values were then used to stratify the patients. A multivariable logistic regression analysis was performed to determine the risk factors for a CPAP indication by PSG using the following variables: a male sex, age ≥ 65 years old, body mass index (BMI) ≥ 25 kg/m^2^, non‐paroxysmal AF (persistent AF and long‐lasting AF), history of heart failure, hypertension, diabetes, vascular disease, ESS ≥ 11, left ventricular diastolic diameter (LVDd) ≥ 55 mm, left atrial diameter (LAD) ≥ 40 mm, and EF < 50% on the echocardiogram. All statistical analyses were performed using JMP13.2.1 software (SAS Institute, Inc.).

## 3 RESULTS

### 3.1 Overall description of the sample

Overall, 464 patients were enrolled. The baseline characteristics are listed in Table 1. The mean age was 64.9 ± 10.6 years, mean BMI 24.6 ± 3.5 kg/m^2^, and 76.5% of the patients were men. The prevalence of paroxysmal AF was 45%. The mean CHADS_2_ score and CHA_2_DS_2_‐VASc score were 1.17 and 2.00, respectively. The mean ESS scale was 6.8 ± 4.5.

**TABLE 1:** Baseline characteristics of the overall patients.

Characteristic	Overall (*N* = 464)
Age, years	64.9 ± 10.6
Male sex, *n* (%)	355 (76.5)
Height, cm	167.1 ± 9.0
Body weight, kg	69.1 ± 13.2
Body mass index, kg/m^2^	24.6 ± 3.5
AF type	
Paroxysmal, *n* (%)	209 (45.0)
Persistent, *n* (%)	185 (39.9)
Long lasting, *n* (%)	70 (15.1)
History of heart failure, *n* (%)	66 (14.2)
Hypertension, *n* (%)	265 (57.1)
Diabetes, *n* (%)	70 (15.1)
Ischemic stroke, *n* (%)	26 (5.6)
Vascular disease, *n* (%)	23 (5.0)
CHA2DS2‐VASc score	
0 or 1 *n* (%)	179 (38.6)
2 or 3 *n* (%)	222 (47.8)
≥4, *n* (%)	63 (13.6)
Mean	2.00
Echocardiographic parameter	
LVDd, mm	47.7 ± 5.0
LVEF, %	63.5 ± 10.5
LAD, mm	40.8 ± 6.1
Epworth sleepiness scale	6.8 ± 4.5

*Note*: All data indicate the mean ± standard deviation unless otherwise indicated.

Abbreviations: AF, atrial fibrillation; LAD, left atrial dimension; LVDd, left ventricular dilated diameter; LVEF, left ventricular ejection fraction.

### 3.2 Distribution of AHI

**FIGURE 1:**
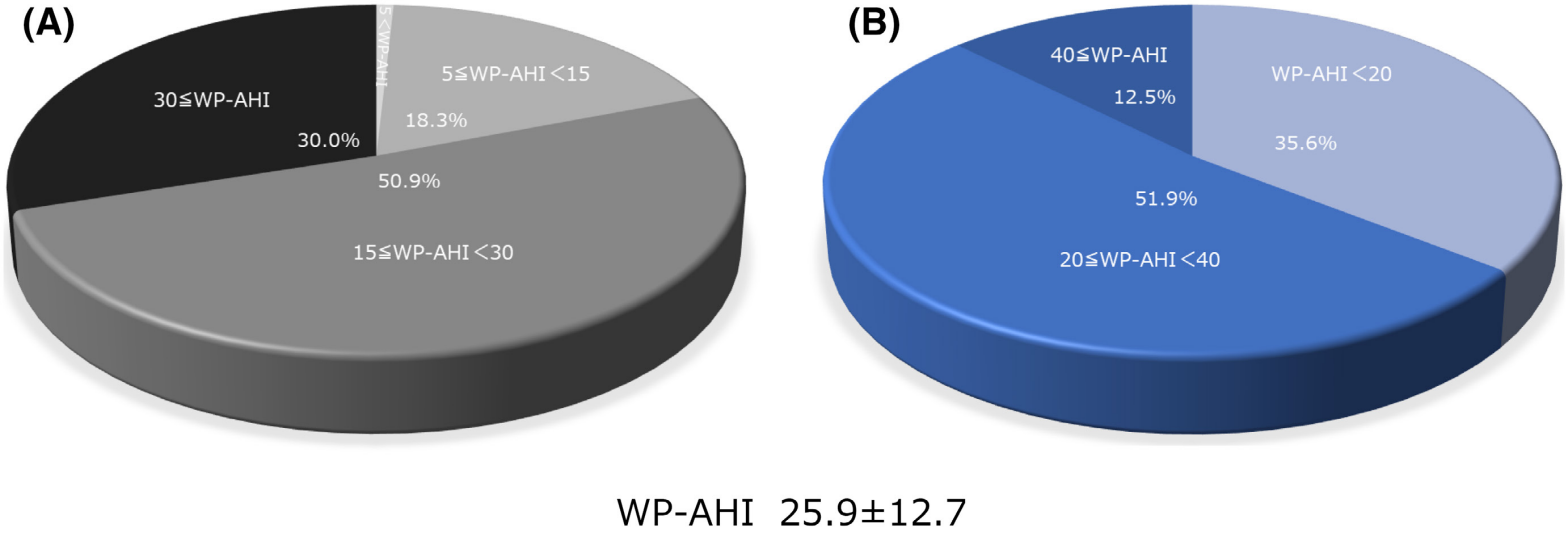
AHI assessed by a home sleep apnea test using Watch PAT tonometry (WP‐AHI). (A) The assessment according to 5 < WP‐AHI, 5 ≤ WP‐AHI < 15, 15 ≤ WP‐AHI < 30, and 30 ≤ WP‐AHI. (B) The assessment according to 20 < WP‐AHI, 20 ≤ WP‐AHI < 40, and 40 ≤ WP‐AHI.

**FIGURE 2:**
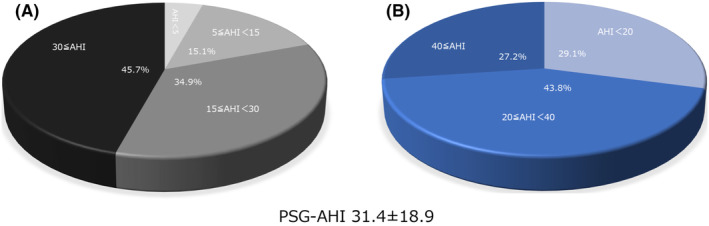
AHI assessed by Polysomnography (PSG‐AHI). (A) The assessment according to 5 < AHI, 5 ≤ AHI < 15, 15 ≤ AHI < 30, and 30 ≤ AHI. (B) The assessment according to 20 < AHI, 20 ≤ AHI < 40, and 40 ≤ AHI.

The distribution of the WP‐AHI and PSG‐AHI is shown in Figure 1 and 2. The mean and median WP‐AHI were 25.9 ± 12.7 and 23.4 (16.7–32.3), respectively. The number of patients with a WP‐AHI <5 (no sleep apnea), 5 ≤ WP‐AHI < 15 (mild sleep apnea), 15 ≤ WP‐AHI < 30 (moderate sleep apnea), and WP‐AHI ≥ 30 (severe sleep apnea) was 4 (0.9%), 85 (18.3%), 236 (50.9%), and 139 (30.0%), respectively (Figure 1A). The number of patients with a WP‐AHI < 20, 20 ≤ WP‐AHI < 40, and WP‐AHI ≥ 40 included 165 (35.6%), 241 (51.9%), and 58 (12.5%), respectively (Figure 1B).

The mean and median PSG‐AHI were 31.4 ± 18.9 and 26.9 (17.1–43.4), respectively. The number of patients with an PSG‐AHI < 5, 5 ≤ PSG‐AHI < 15, 15 ≤ PSG‐AHI < 30, and PSG‐AHI ≥ 30 was 20 (4.3%), 70 (15.1%), 162 (34.9%), and 212 (45.7%), respectively (Figure 2A). The number of patients with an PSG‐AHI < 20, 20 ≤ PSG‐AHI < 40, and PSG‐AHI ≥ 40 was 135 (29.1%), 203 (43.8%), and 126 (27.2%) patients, respectively (Figure 2B).

### 3.3 Correlation between the WP‐AHI and PSG‐AHI

**FIGURE 3:**
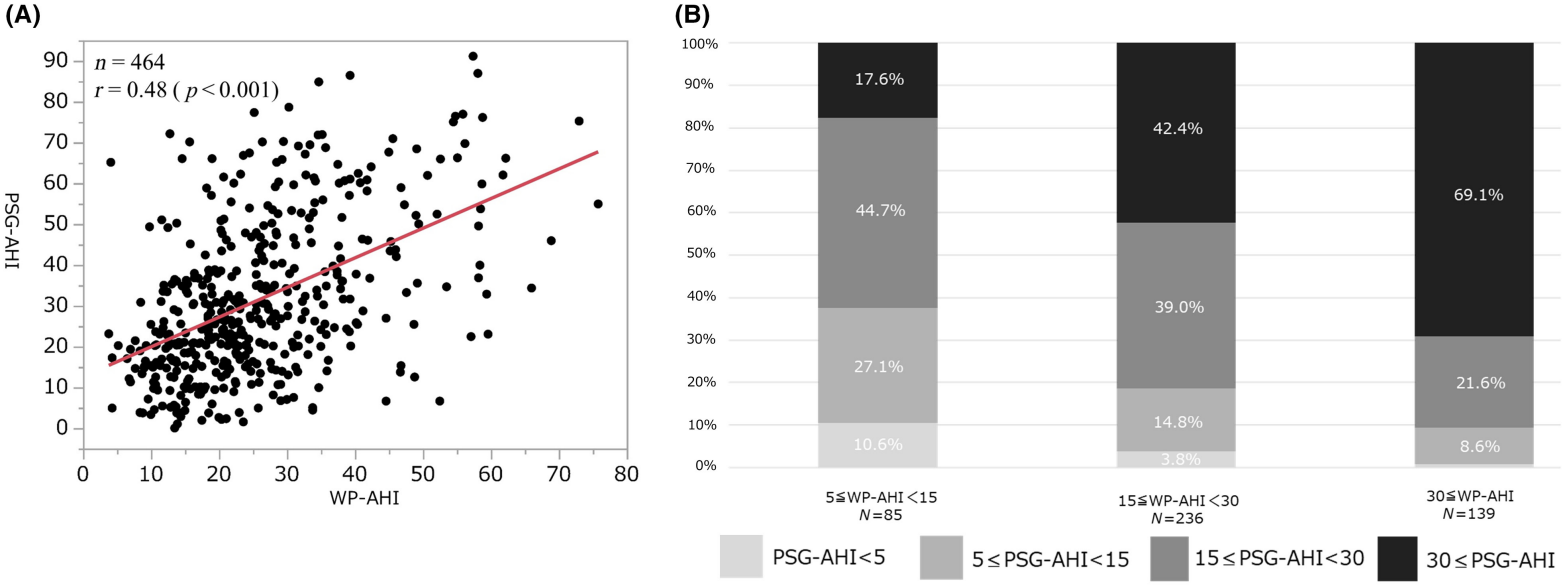
(A) Correlation of the PSG‐AHI with the WP‐AHI and (B) the severity of the sleep apnea (PSG‐AHI) according to the WP‐AHI.

There was a correlation between the WP‐AHI and PSG‐AHI (*r* = .48, *p* < .001) (Figure 3A). Among 325 patients with a WP‐AHI < 30, 116 patients (35.7%) exhibited a PSG‐AHI ≥ 30. Among 139 patients with a WP‐AHI ≥ 30, 96 (69.1%) exhibited a PSG‐AHI ≥ 30 (Figure 3B).

### 3.4 The risk factors for a CPAP indication

**FIGURE 4:**
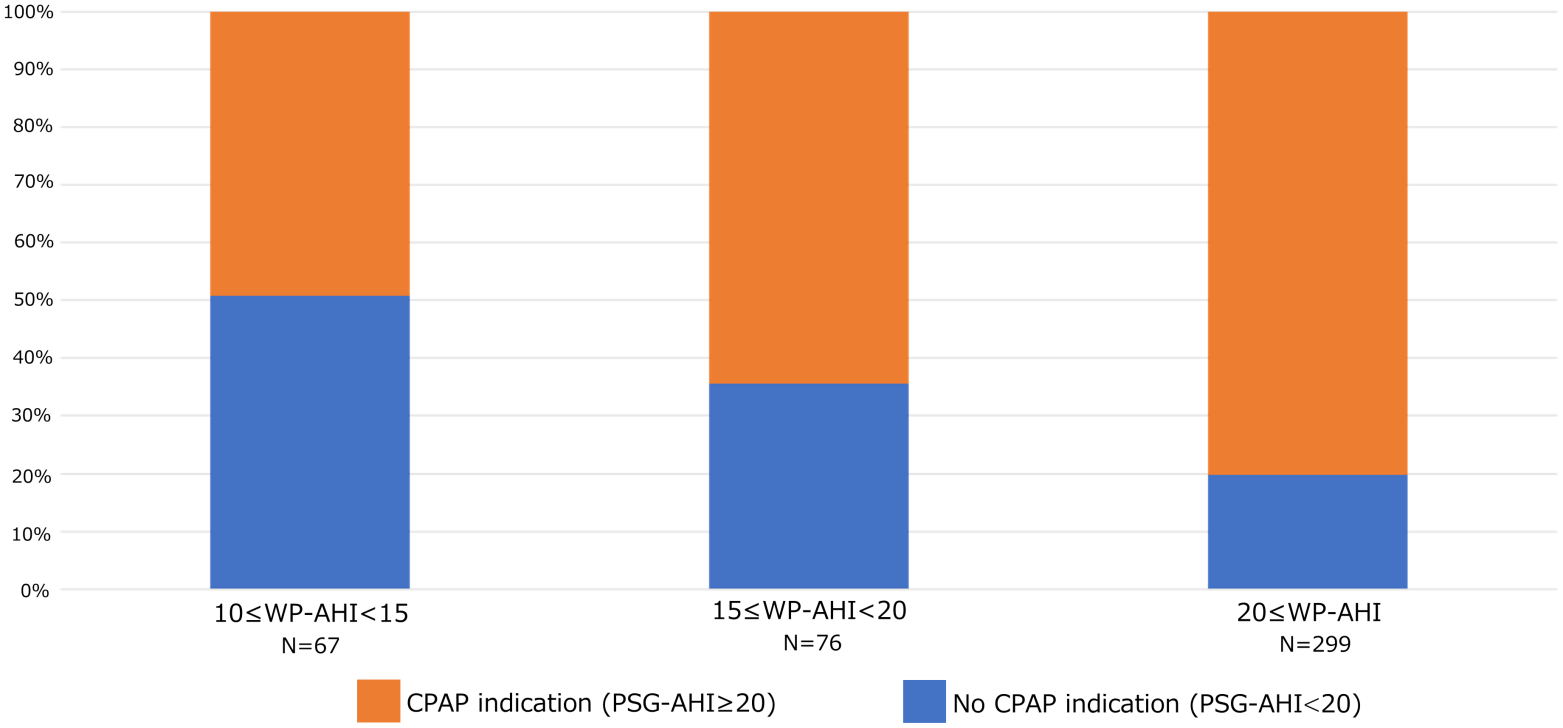
Indication for CPAP (PSG‐AHI ≥ 20) using PSG according to the severity assessed using the Watch PAT (WP‐AHI).

**FIGURE 5:**
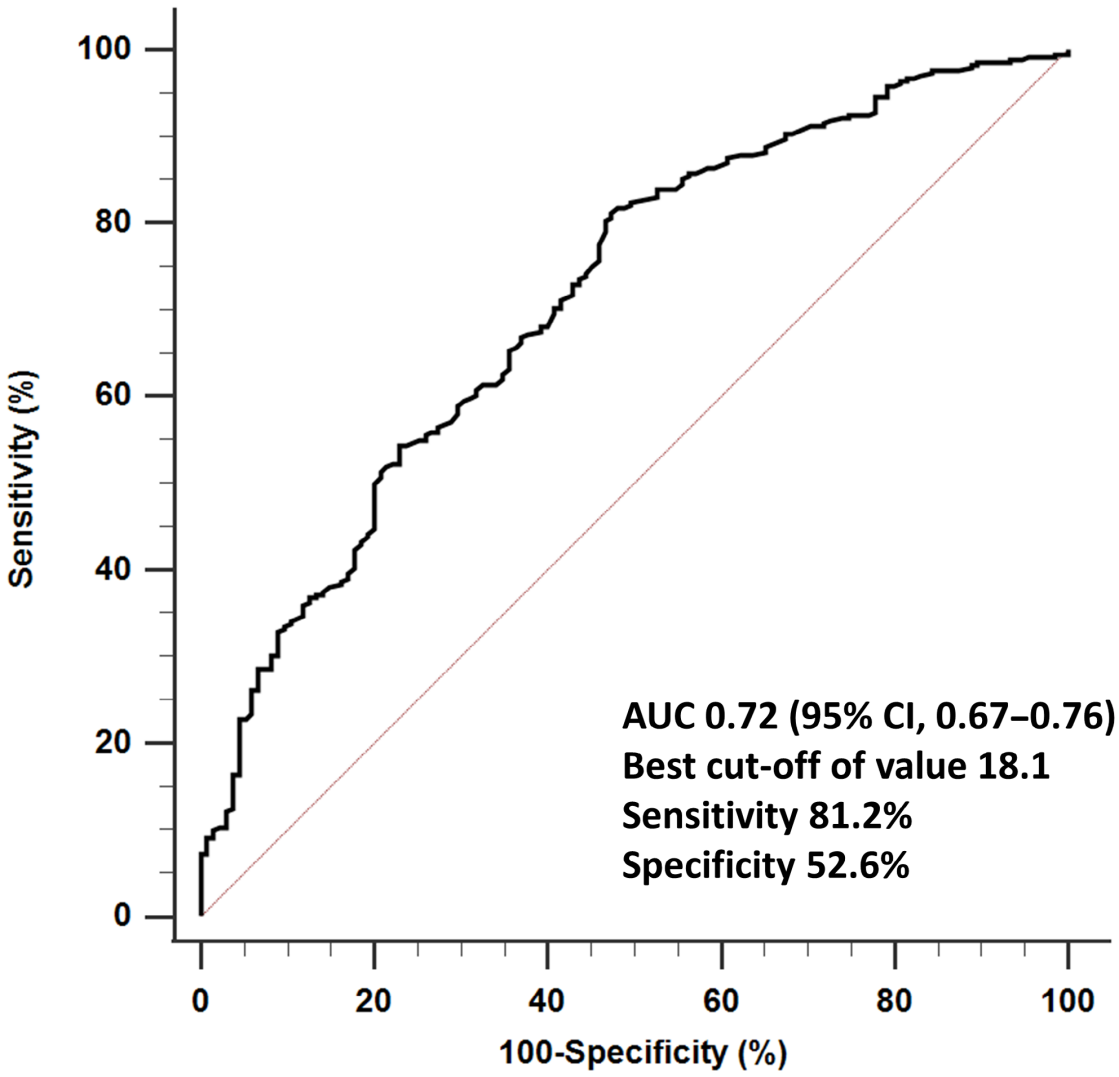
Receiver operating characteristic curves of the WP‐AHI for a PSG‐AHI ≥ 20 (CPAP indication).

**FIGURE 6:**
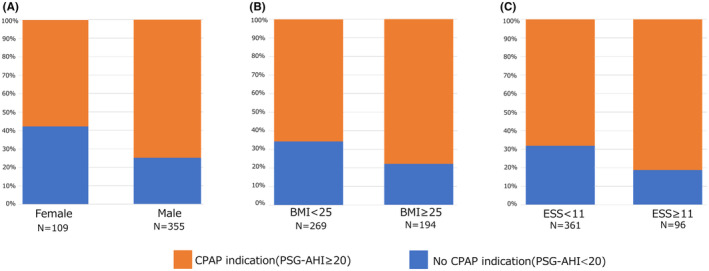
CPAP indication (PSG‐AHI ≥ 20) according to sex (A), obesity (B), and Epworth Sleepiness Scale (ESS) (C).

In patients with a 10 ≤ WP‐AHI < 15 (*n* = 67), 15 ≤ WP‐AHI < 20 (*n* = 76), and WP‐AHI ≥ 20 (*n* = 299), 33 (49.3%), 49 (64.5%), and 240 (80.3%) patients had a CPAP indication assessed by PSG (20 ≤ PSG‐AHI), respectively (Figure 4). An ROC curve analysis revealed that an optimal WP‐AHI cut‐off value of 18.1 could predict a CPAP indication (PSG‐AHI ≥ 20) with an AUC of 0.72 (95% CI 0.67–0.76) (Figure 5). A WP‐AHI ≥ 18.1 (OR, 4.22; 95% CI: 2.63–6.76, *p* < .0001), male sex (OR, 2.27; 95% CI: 1.33–3.89, *p* = .0028), and sleepiness (OR, 2.02; 95% CI: 1.10–3.71, *p* = .024) were significant predictors of a CPAP indication in the multivariate analysis (Table 2). However, the prevalence of a CPAP indication in patients with a female sex, without obesity, and without sleepiness, was 57.8%, 65.8%, and 68.1%, respectively (Figure 6).

**TABLE 2:** Risk factors of a CPAP indication assessed by PSG (PSG‐AHI ≥ 20). Univariate and multivariate analysis.

	Univariate *p* values	OR	95% CI	Multivariate *p* values	OR	95% CI
WP‐AHI ≥ 18.1	<.0001	4.78	3.09–7.39	<.0001	4.22	2.63–6.76
Male	.0007	2.18	1.39–3.42	.0028	2.27	1.33–3.89
Age ≥ 65	.54	1.14	0.76–1.70	.19	1.40	0.85–2.30
BMI ≥ 25	.0052	1.83	1.20–2.78	.43	1.22	0.74–2.01
Non‐paroxysmal AF	.81	1.05	0.70–1.57	.21	0.72	0.44–1.20
History of heart failure	.35	1.33	0.73–2.43	.44	1.34	0.64–2.78
Hypertension	.022	1.60	1.07–2.40	.16	1.41	0.88–2.26
Diabetes	.13	1.61	0.87–2.96	.19	1.58	0.79–3.17
Vascular disease	.54	0.76	0.31–1.84	.075	0.40	0.15–1.10
LVDd ≥ 55	.046	2.48	1.02–6.05	.15	2.31	0.75–7.14
LAD ≥ 40	.009	1.72	1.14–2.57	.23	1.37	0.82–2.29
LVEF < 50	.12	1.82	0.85–3.88	.96	0.98	0.40–2.41
ESS ≥ 11	.013	2.03	1.16–3.54	.024	2.02	1.10–3.71

Abbreviations: AF, atrial fibrillation; BMI, body mass index; ESS, Epworth sleepiness scale; LAD, left atrial dimension; LVDd, left ventricular dilated diameter; LVEF, left ventricular ejection fraction.

## 4 DISCUSSION

### 4.1 Main findings

The present study to compare the WP‐AHI with the PSG‐AHI for unscreened sleep apnea patients receiving CA of AF revealed the following findings. (1) Although there was a correlation between the WP‐AHI and PSG‐AHI, the PSG‐AHI was higher than the WP‐AHI. (2) While only 12.5% of the patients were indicated for CPAP treatment assessed only by HSAT, 70.9% of the patients were indicated for CPAP assessed by PSG according to the current Japanese health insurance system. (3) The best cut‐off value of the WP‐AHI was 18.1 to predict a CPAP indication assessed using PSG. The risk factors of a CPAP indication assessed by PSG were a WP‐AHI ≥ 18.1, male sex, and ESS ≥ 11. However, the majority of the female and non‐sleepiness patients did have a CPAP indication.

### 4.2 Sleep apnea in AF patients receiving CA

Sleep apnea is a risk factor for AF recurrence after CA. Several meta‐analyses in some nonrandomized studies have established that CPAP treatment for severe OSA is associated with higher AF‐free survival rates in AF patients undergoing CA.^6^ The WP is a type of HSAT that allows an automatic calculation of the scoring. Previous studies showed a strong correlation between the AHI using the WP and the simultaneously recorded AHI using PSG in patients with cardiovascular diseases including AF.^14^ We previously reported that almost all patients with AF could self‐administer this device without any assistance and succeeded in obtaining their AHI data. The prevalence of sleep apnea was 88.6% and 53.2% of them had moderate or severe sleep apnea (AHI ≥ 15) in unscreened sleep apnea patients prior to the CA of AF. However, many of the moderate or severe sleep apnea patients with AF did not have sleepiness, so we may have overlooked their sleep apnea unless we performed the HSAT prior to the CA.^9^ That sleep apnea detection before the CA would have led the operators to avoid deep sedation during the pulmonary vein isolation and to more easily obtain a better catheter stability.

As a result of a routine HSAT in AF patients prior to the CA, we should consider carrying out PSG in moderate or severe sleep apnea patients after the CA. There are two reasons for this as follows: (1) the indication for CPAP treatment is an AHI ≥ 20 assessed using PSG or an AHI ≥ 40 assessed by HSAT according to the current Japanese health insurance system.^15^ Therefore, patients with an AHI < 40 assessed by HSAT are not indicated for CPAP treatment without PSG even if they have moderate or severe sleep apnea assessed by HSAT. (2) Some studies have indicated that conversion from AF to SR reduces the AHI following CA or cardioversion.^16^

### 4.3 Weak correlation between the WP‐AHI and PSG‐AHI

OSA screening using HSAT before the CA is more cost‐effective than when using PSG in AF patients.^17^ Many studies using the WP have excluded patients with arrhythmias due to the potential effect of the arrhythmias on the peripheral arterial tonometry amplitude and rate changes. Tauman R et al. showed that a strong correlation was found between the PSG‐AHI and WP‐AHI (*r* = .80, *p* < .0001), and the presence of AF during the night did not decrease the precision of the WP‐AHI when full in‐lab PSG and WP were conducted simultaneously in patients with AF.^18^ We revealed that the AHI obtained by PSG in our institution after the CA was higher than that of the WP at home before the CA. More than half of the mild sleep apnea patients assessed by the WP were diagnosed with moderate or severe sleep apnea by PSG, and 42% of the moderate sleep apnea patients assessed by the WP were diagnosed with severe sleep apnea by PSG. There were 3 possible reasons why a weak correlation was found between the WP‐AHI and PSG‐AHI in the current study which are as follows: (1) the difference in the tools used, (2) rhythm differences, and (3) the night‐to‐night variability.

1. In the aforementioned trial, WP‐AHI was well correlated with the PSG‐AHI when performing the WP and PSG simultaneously in AF patients.^18^ The number of study subjects was relatively small (*n* = 101) and did not have a perfect correlation (*r* = .80).
2. Some studies showed that conversion from AF to sinus rhythm reduces the AHI following CA or cardioversion. Naruse Y et al. described a decrease in the median AHI from 22 to 15 following a successful ablation, while almost all patients with moderate or severe sleep disorder breathing before CA still had sleep disorder breathing after RFCA. In their study, all patients (*n* = 25) underwent polysomnography 1 day before and 1 week after CA.^19^ In our current study, the majority of the patients with paroxysmal AF (*n* = 209) underwent WP and PSG during sinus rhythm, while almost all non‐paroxysmal AF patients (*n* = 255) underwent the WP during an AF rhythm and PSG during SR rhythm. However, there was a similar correlation between the WP‐AHI and PSG‐AHI in terms of the AF type (paroxysmal AF vs. non‐paroxysmal AF, *r* = .47 vs. *r* = .49).
3. There is a considerable number of studies assessing the night‐to‐night variability in the AHI.^20^ Roeder M et al. evaluated the night‐to‐night variability of respiratory events in adults with suspected or already diagnosed OSA who underwent more than one diagnostic sleep study. They revealed that 41% of all participants exhibited changes in the AHI of >10/h from night to night and 49% of them changed their OSA severity class.^21^ The sleep position and sleep stage could play an important role in the night‐to‐night variability because a supine position and rapid eye movement sleep reduce the effectiveness of the compensatory mechanisms to counteract an upper airway collapse during OSA.^22^ Experts recommend PSG for the diagnosis of OSA if a single HSAT is inconclusive.^10^

### 4.4 PSG eligibility after the HSAT results in AF patients

A diagnosis of OSA requires the patient to have (1) reported nocturnal breathing disturbances (snoring or breathing pauses during sleep) or symptoms of daytime sleepiness or fatigue and (2) an AHI ≥ 5. OSA is also diagnosed if the AHI is ≥15 even in the absence of symptoms.^10^ Regardless of using PSG or HSAT, a CPAP indication is based on an AHI ≥ 15 without symptoms or an AHI ≥ 5 with documented symptoms including excessive daytime sleepiness or documented cardiovascular diseases including AF in the United States.^23^ On the other hand, an indication for CPAP treatment is defined as an AHI ≥ 20 assessed using PSG or an AHI ≥ 40 assessed using HSAT according to the current Japanese health insurance system. Although sleep apnea is an independent risk factor for the development of cardiovascular diseases such as hypertension, heart failure, and coronary artery disease in addition to AF, a CPAP indication for OSA has nothing to do with the prevalence of cardiovascular diseases.^24^ AF patients with an AHI < 40 using HSAT need an additional PSG to determine the CPAP indication. PSG usually requires being admitted for one night. It is time‐consuming, and costly as compared to HSAT. Further, HSAT is a more preferable tool than PSG to prevent infections under the recent COVID 19 epidemic circumstances. In the real clinical setting, a certain proportion of AF patients are reluctant to receive PSG after a diagnosis of sleep apnea by HSAT. The revised guidelines regarding a CPAP indication by HSAT should be discussed especially in AF patients to improve their rhythm control.

However, under the current system in Japan, we should strongly recommend PSG for determining a CPAP indication in AF patients after a diagnosis of moderate or severe OSA by HSAT, because our results revealed that only 12.5% of patients were indicated for CPAP treatment by HSAT, while 70.9% were indicated for CPAP treatment by PSG. Moreover, we should consider carrying out PSG even when the patients are diagnosed with mild sleep apnea by HSAT because half of the patients with a 10 ≤ WP‐AHI < 15 and 64.5% of the patients with a 15 ≤ WP‐AHI < 20 had a CPAP indication by PSG. Even if they have only mild sleep apnea by HSAT, we should recommend PSG, especially in males or patients with daytime sleepiness.

### 4.5 Study limitations

The main limitations of this study were associated with the retrospective, observational design. We believe, however, the findings obtained in our clinical practice compare favorably with those of a well‐organized prospective study because we collected sequential data on unscreened sleep apnea patients with AF who underwent the WP and PSG during a relatively short period, and the sample size was large (*n* = 464). Second, CA might have influenced our results because the WP was performed before the CA, and the patients received PSG after the CA. Third, the WP demonstrated a high degree of correlation of the AHI with the PSG, but the evidence of the accuracy in patients with AF or in those who have central sleep apnea is not adequate. Fourth, this study was based on a single‐center experience. To ensure that AF patients should receive PSG after the HSAT, our results should be confirmed by a multicenter study.

## 5 CONCLUSIONS

In AF patients receiving catheter ablation, there was a significant variation between the WP‐AHI and PSG‐AHI. About one‐third of the patients with mild or moderate sleep apnea using WP was diagnosed with severe sleep apnea evaluated by PSG. Even if routine sleep apnea screening using HSAT before CA results in only mild sleep apnea, PSG should be considered especially in patients that are male or have daytime sleepiness, in order to determine an indication for CPAP treatment.

## CONFLICT OF INTEREST STATEMENT

N. Tanaka has received honoraria from Johnson and Johnson KK, Bayer, and Boehringer Ingelheim. The other authors declare no conflicts of interest.

## ETHICS APPROVAL STATEMENT

The present study was approved by Sakurabashi Watanabe Hospital (Reference number. 21–89).

## PATIENT CONSENT STATEMENT

Informed consent was obtained in the form of opt‐out on the website. Those who rejected were excluded.

## CLINICAL TRIAL REGISTRATION

N/A.

## References

[1] Schnabel RB , Yin X , Gona P , Larson MG , Beiser AS , McManus DD , et al. 50 year trends in atrial fibrillation prevalence, incidence, risk factors, and mortality in the Framingham Heart Study: a cohort study. Lancet. 2015;386(9989):154–62. 10.1016/S0140-6736(14)61774-8 25960110 PMC4553037

[2] Kasai T , Floras JS , Bradley TD . Sleep apnea and cardiovascular disease: a bidirectional relationship. Circulation. 2012;126:1495–510. 22988046 10.1161/CIRCULATIONAHA.111.070813

[3] Linz D , McEvoy RD , Cowie MR , Somers VK , Nattel S , Lévy P , et al. Associations of obstructive sleep apnea with atrial fibrillation and continuous positive airway pressure treatment: a review. JAMA Cardiol. 2018;3:532–40. 29541763 10.1001/jamacardio.2018.0095

[4] Harmon EK , Stafford P , Ibrahim S , Cho Y , Mazimba S , Bilchick K , et al. Atrial fibrillation is associated with central sleep apnea in clinic patients undergoing diagnostic polysomnography. J Arrhythm. 2020;36(6):991–6. 10.1002/joa3.12427 33335615 PMC7733563

[5] Cadby G , McArdle N , Briffa T , Hillman DR , Simpson L , Knuiman M , et al. Severity of OSA is an independent predictor of incident atrial fibrillation hospitalization in a large sleep‐clinic cohort. Chest. 2015;148(4):945–52. 10.1378/chest.15-0229 25927872

[6] Li L , Wang ZW , Li J , Ge X , Guo LZ , Wang Y , et al. Efficacy of catheter ablation of atrial fibrillation in patients with obstructive sleep apnoea with and without continuous positive airway pressure treatment: a meta‐analysis of observational studies. Europace. 2014;16(9):1309–14. 10.1093/europace/euu066 24696222

[7] Fein AS , Shvilkin A , Shah D , Haffajee CI , Das S , Kumar K , et al. Treatment of obstructive sleep apnea reduces the risk of atrial fibrillation recurrence after catheter ablation. J Am Coll Cardiol. 2013;62(4):300–5. 10.1016/j.jacc.2013.03.052 23623910

[8] Qureshi WT , Nasir UB , Alqalyoobi S , O'Neal WT , Mawri S , Sabbagh S , et al. Meta‐Analysis of continuous positive airway pressure as a therapy of atrial fibrillation in obstructive sleep apnea. Am J Cardiol. 2015;116:1767–73. 26482182 10.1016/j.amjcard.2015.08.046

[9] Tanaka N , Tanaka K , Hirao Y , Okada M , Ninomiya Y , Yoshimoto I , et al. Home sleep apnea test to screen patients with atrial fibrillation for sleep apnea prior to catheter ablation. Circ J. 2021;85(3):252–60. 10.1253/circj.CJ-20-0782 33298643

[10] Kapur VK , Auckley DH , Chowdhuri S , Kuhlmann DC , Mehra R , Ramar K , et al. Clinical practice guideline for diagnostic testing for adult obstructive sleep apnea: an American Academy of sleep medicine clinical practice guideline. J Clin Sleep Med. 2017;13(3):479–504. 10.5664/jcsm.6506 28162150 PMC5337595

[11] Yalamanchali S , Farajian V , Hamilton C , Pott TR , Samuelson CG , Friedman M . Diagnosis of obstructive sleep apnea by peripheral arterial tonometry: meta‐analysis. JAMA Otolaryngol Head Neck Surg. 2013;139:1343–50. 24158564 10.1001/jamaoto.2013.5338

[12] Inoue K , Tanaka N , Ikada Y , Mizutani A , Yamamoto K , Matsuhira H , et al. Characterizing clinical outcomes and factors associated with conduction gaps in VISITAG SURPOINT‐guided catheter ablation for atrial fibrillation. J Arrhythm. 2021;37(3):574–83. 10.1002/joa3.12544 34141010 PMC8207404

[13] Johns MW . A new method for measuring daytime sleepiness: the Epworth Sleepiness Scale. Sleep. 1991;14:540–5. 1798888 10.1093/sleep/14.6.540

[14] Kasai T , Takata Y , Yoshihisa A , Takeishi Y , Chin K , Ando SI , et al. Comparison of the apnea‐hypopnea index determined by a peripheral arterial tonometry‐based device with that determined by polysomnography ‐ Results from a multicenter study. Circ Rep. 2020;2(11):674–81. 10.1253/circrep.CR-20-0097 33693194 PMC7937496

[15] Akashiba T , Inoue Y , Uchimura N , Ohi M , Kasai T , Kawana F , et al. Sleep Apnea Syndrome (SAS) clinical practice guidelines 2020. Respir Investig. 2022;60(1):3–32. 10.1016/j.resinv.2021.08.010 34986992

[16] Fox H , Bitter T , Horstkotte D , Oldenburg O . Cardioversion of atrial fibrillation or atrial flutter into sinus rhythm reduces nocturnal central respiratory events and unmasks obstructive sleep apnoea. Clin Res Cardiol. 2016;105(5):451–9. 10.1007/s00392-015-0940-2 26552903

[17] Kawakami H , Saito M , Kodera S , Fujii A , Nagai T , Uetani T , et al. Cost‐effectiveness of obstructive sleep apnea screening and treatment before catheter ablation for symptomatic atrial fibrillation. Circ Rep. 2020;2(9):507–16. 10.1253/circrep.CR-20-0074 33693276 PMC7819651

[18] Tauman R , Berall M , Berry R , Etzioni T , Shrater N , Hwang D , et al. Watch‐PAT is useful in the diagnosis of sleep apnea in patients with atrial fibrillation. Nat Sci Sleep. 2020;12:1115–21. 10.2147/NSS.S278752 33299372 PMC7721305

[19] Naruse Y , Tada H , Satoh M , Yanagihara M , Tsuneoka H , Hirata Y , et al. Radiofrequency catheter ablation of persistent atrial fibrillation decreases a sleep‐disordered breathing parameter during a short follow‐up period. Circ J. 2012;76:2096–103. 22664722 10.1253/circj.cj-12-0014

[20] Gouveris H , Selivanova O , Bausmer U , Goepel B , Mann W . First‐night‐effect on polysomnographic respiratory sleep parameters in patients with sleep‐disordered breathing and upper airway pathology. Eur Arch Otorhinolaryngol. 2010;267:1449–53. 20127101 10.1007/s00405-010-1205-3

[21] Roeder M , Bradicich M , Schwarz EI , Thiel S , Gaisl T , Held U , et al. Night‐to‐night variability of respiratory events in obstructive sleep apnoea: a systematic review and meta‐analysis. Thorax. 2020;75(12):1095–102. 10.1136/thoraxjnl-2020-214544 32792405

[22] Younes M . Contributions of upper airway mechanics and control mechanisms to severity of obstructive apnea. Am J Respir Crit Care Med. 2003;168(6):645–58. 10.1164/rccm.200302-201OC 12773321

[23] Yeghiazarians Y , Jneid H , Tietjens JR , Redline S , Brown DL , El‐Sherif N , et al. Obstructive sleep apnea and cardiovascular disease: a scientific statement from the American Heart Association. Circulation. 2021;144(3):e56–67. 10.1161/CIR.0000000000000988 34148375

[24] Javaheri S , Parker TJ , Liming JD , Corbett WS , Nishiyama H , Wexler L , et al. Sleep apnea in 81 ambulatory male patients withstable heart failure: types and their prevalences, consequences, and presentations. Circulation. 1998;97:2154–9. 9626176 10.1161/01.cir.97.21.2154

